# Adaptive evolution of synchronous egg-hatching in compensation for the loss of parental care

**DOI:** 10.1098/rspb.2018.1452

**Published:** 2018-08-29

**Authors:** Benjamin J. M. Jarrett, Darren Rebar, Hannah B. Haynes, Miranda R. Leaf, Chay Halliwell, Rachel Kemp, Rebecca M. Kilner

**Affiliations:** 1 Department of Zoology, University of Cambridge, Downing Street, Cambridge CB2 3EJ, UK; 2 Department of Biological Sciences, Emporia State University, Emporia, KS 66801, USA

**Keywords:** hatching asynchrony, experimental evolution, sibling cooperation, sibling rivalry, adaptation

## Abstract

Interactions among siblings are finely balanced between rivalry and cooperation, but the factors that tip the balance towards cooperation are incompletely understood. Previous observations of insect species suggest that (i) sibling cooperation is more likely when siblings hatch at the same time, and (ii) this is more common when parents provide little to no care. In this paper, we tested these ideas experimentally with the burying beetle, *Nicrophorus vespilloides*. Burying beetles convert the body of a small dead vertebrate into an edible nest for their larvae, and provision and guard their young after hatching. In our first experiment, we simulated synchronous or asynchronous hatching by adding larvae at different intervals to the carrion-breeding resource. We found that ‘synchronously’ hatched broods survived better than ‘asynchronously’ hatched broods, probably because ‘synchronous hatching’ generated larger teams of larvae, that together worked more effectively to penetrate the carrion nest and feed upon it. In our second experiment, we measured the synchronicity of hatching in experimental populations that had evolved for 22 generations without any post-hatching care, and control populations that had evolved in parallel with post-hatching care. We found that larvae were more likely to hatch earlier, and at the same time as their broodmates, in the experimental populations that evolved without post-hatching care. We suggest that synchronous hatching enables offspring to help each other when parents are not present to provide care. However, we also suggest that greater levels of cooperation among siblings cannot compensate fully for the loss of parental care.

## 1. Introduction

Offspring that develop alongside each other in the same nursery commonly compete for limited resources such as food [1]. Nevertheless, there is growing empirical evidence from a range of taxa that siblings can also cooperate to promote each other's fitness. For example, offspring may collectively defend resources for relatives from attack by unrelated rivals [2]. Or they may work as a collective to procure more resources from provisioning adults [3,4]. Sometimes they even share food directly with one another [5,6]. Interactions among siblings are thus finely balanced between conflict and cooperation [1,7,8] and a key problem is to understand the factors that cause a transition between them.

Parents set the stage in determining whether there is more likely to be conflict or cooperation among siblings. Extensive studies of different bird species have tested the suggestion that parents can modulate the extent of rivalry among their young through hatching asynchrony (reviewed in [1,9,10]). By starting to incubate her clutch before completing egg-laying, a mother bird can cause her offspring to hatch asynchronously. This establishes a size hierarchy within the brood, and associated asymmetries among offspring in their ability to compete for food delivered to the nest by parents. Although the function of these competitive asymmetries, and the key beneficiaries of hatching asynchrony, are still much debated, there is now overwhelming evidence that hatching asynchrony plays an important role in modulating the extent of competition among offspring (e.g. [1,9]).

Here, we investigate whether parents could likewise facilitate greater levels of *cooperation* among their offspring by hatching them more *synchronously*. This possibility is suggested by circumstantial evidence from insect species without parental care. By hatching synchronously, offspring might collectively increase their survival by diluting the risk of attack by predators [11]. Or they might more effectively thwart predators through large, coordinated aggressive displays [12,13]. Synchronized hatching might also benefit offspring because collectively offspring are better able to acquire resources. For example, larger numbers of *Brevicoryne brassicae* aphids can more effectively extract nutrients from the phloem of plants [14].

In species with facultative care, or a highly variable supply of parental care, the extent of larval cooperation inversely, and flexibly, varies with the level of parental care supplied. For example, in the European earwig *Forficula auricularia*, the incidence of food sharing among nymphs is greater when maternal food provisioning is lower [15]. Recent work on the burying beetle *Nicrophorus vespilloides* provides further evidence of a dynamic interplay between parental care and larval cooperation. When parents tend the brood, there is a trade-off between brood size and larval size, implying that competition for resources increases with increasing larval density [16]. When parents are absent, however, there is a positive association between brood size and larval mass at low larval densities, implying that at these low larval densities, each extra offspring can somehow help its siblings gain mass. Since larval mass is positively correlated with fitness [17], this means each larva is effectively acting cooperatively [16]. Experiments on the subsocial burrower bug *Adomerus triguttulus* explicitly link the extent of hatching synchrony to the nature of larval interactions and the supply of maternal care. In this species, maternal care is facultative and, when mothers are absent, earlier hatched offspring eat their unhatched siblings. Mothers can counteract this problem by inducing synchronous hatching [18] to reduce competition among siblings [19].

The picture that emerges from these examples is that (i) parents can increase the extent to which their offspring cooperate by ensuring that they hatch synchronously (i.e. within a narrow timeframe), and (ii) greater levels of offspring cooperation are expected in species that exhibit either lower levels of parental care, or none at all. Therefore, in species with facultative parental care we predict that: (i) an increase in the extent of hatching synchrony within the brood should promote offspring fitness in the absence of parental care, and (ii) after sustained exposure to no post-hatching parental care, we should see an evolved increase in the degree to which hatching is synchronized within each brood.

We tested these predictions with experiments on the burying beetle *N. vespilloides*. To test prediction (i), we performed an experimental manipulation on a single generation of burying beetles, to test whether synchronous hatching promotes larval survival in the absence of parental care. To test prediction (ii) we used experimental populations of burying beetles that had evolved without post-hatching parental care for 22 generations [20,21]. We determined whether larvae within broods from these No Care populations were now more likely to hatch at the same time as each other than larvae from experimental populations, kept in parallel, which had been continuously exposed to parental care.

## 2. Methods

### (a) Study species

Burying beetles breed on small vertebrate carrion [22,23]. To make the carcass into an edible resource for their offspring, parent beetles shave off the fur or feathers, mould the flesh into a ball, cover it in an anti-microbial fluid and bury it. Eggs are laid in the soil surrounding the carcass, and after the larvae hatch, they crawl to the carcass. Parents bite small incisions in the flesh to allow the larvae to penetrate the carcass and the larvae congregate within, where they are fed by their parents. The larvae can also self-feed. Parental care increases the fitness of larvae [24], but larvae can survive without post-hatching parental care [20]. Approximately five days after the larvae hatch, the larvae disperse from the few remnants of the carcass, to pupate in the soil. It is at this stage that we weighed larvae in our experiments.

### (b) Experiment 1: Is hatching ‘synchrony’ adaptive in the absence of parental care?

All experiments were conducted on laboratory populations of burying beetles maintained in the Department of Zoology at the University of Cambridge, UK. General details of their husbandry are given in ref [25]. When individuals reached sexual maturity, two weeks after emerging as adults, we paired unrelated males and females (*N* = 70 pairs) from a single stock population that had always experienced parental care, and that was independent of the experimental populations described below. Each pair was given a freshly thawed mouse carcass (7–17 g), placed in a breeding box (17 × 12 × 6 cm) lined with soil, and kept in a cupboard to simulate underground conditions.

After 53 h, we removed the parent beetles [20,21]. The carcasses that the parents had prepared for reproduction were placed in fresh breeding boxes, lined with soil. We checked the prepared carcasses for signs of a parental incision, and removed any that bore an incision to ensure that parents were providing no assistance to experimental broods after hatching. These prepared carcasses were then set aside to be used for raising the experimental broods. Meanwhile, every 7 h we checked the original breeding boxes, which each still contained a complete clutch of eggs, for hatching larvae. Newly hatched larvae from different broods were pooled in a petri dish and used haphazardly to create new experimental broods. We deliberately created all the broods from this pool of unrelated individuals to eliminate any potential confounding maternal effects. All experimental broods contained 10 larvae of the same age and similar size. We chose this brood size to ensure that interactions among larvae were more likely to be more cooperative than competitive [16], and it is within the brood range exhibited in our lab-bred population and by wild-caught individuals when they breed in the lab [21].

Larvae were haphazardly assigned to one of two experimental brood treatments: synchronous broods (*N* = 33) in which larvae were all placed on a carcass at the same time, and asynchronous broods (*N* = 35), in which four newly hatched larvae were placed initially on a carcass, followed by two larvae every 7 h thereafter until there were 10 larvae altogether. This sequence of larval addition was designed to mimic the natural sequence of larval arrival at the carcass that can arise as a consequence of hatching asynchrony in *N. vespilloides* [26], and was consistent with the hatching spread of broods described in ref [27]. The synchronous and asynchronous treatments manipulated both hatching skew and hatching spread (see below for a detailed explanation of these terms). In both treatments, larvae were then left to fend for themselves on the carcass and experienced no post-hatching care. Note that there is no evidence from previous work that burying beetles can recognize kin [28]. When the carcass had been consumed, and larvae were starting to disperse away into the soil, approximately five days after placing larvae on the carcass, we counted the number of surviving offspring and weighed each larva to the nearest 0.1 mg.

### (c) Statistical analysis

We analysed the number of surviving larvae using a hurdle model. This approach allowed us to answer two questions: (i) did the brood manipulations affect the incidence of brood failure (where brood failure is defined as 0 larvae surviving to dispersal)? and (ii) for successful broods, where at least one larva survived to dispersal, was the number of surviving larvae affected by our brood manipulations? To do this we implemented a zero-inflated binomial model using the package vgam [29] in R 3.4.3 [30], which used a binomial distribution with logit link function for the zero hurdle model, and a truncated negative binomial distribution with log link function for the count model. We used a two-column response variable of the number of surviving larvae and the number of non-surviving larvae to analyse the effect of treatment on the number of surviving offspring. We included the brood manipulation as a two-level factor (synchronous or asynchronous) and the mass of the carcass as explanatory variables. Larval mass was analysed with a linear model, with the mass of the larva as the response variable, and brood manipulation and the number of surviving larvae as explanatory variables.

### (d) Experiment 2: Has egg-hatching evolved to be more synchronous in the absence of parental care?

We analysed four experimental populations that had been evolving under different regimes of parental care, and which were founded from the same starting population. Further details about these populations are provided in ref [21]. All individuals in Experiment 2 were drawn from Generation 22 of experimental evolution. In two replicate populations (hereafter: Full Care Control), parents had been allowed to stay with their larvae during the period of larval development, at each generation during the preceding 22 generations. We could not force parents to care for their offspring in this treatment, but it is highly likely that they did (see Jarrett *et al.*, in press [31]). In parallel for 22 generations, in the other two replicate populations parents had been removed from the breeding box 53 h after pairing at each generation (hereafter: No Care populations). At the time of parental removal, egg-laying had finished but the larvae had yet to hatch. Therefore these larvae did not receive any post-hatching care.

Seventeen days after their emergence as adults, when individuals were sexually mature, we paired 30 males and females within each population (*N* = 120 pairs in total). Each pair was placed in a separate breeding box with moist soil and a thawed carcass. Pairs were haphazardly assigned a carcass from one of two size classes, small (8–9 g) or medium (16–17 g), yielding 15 pairs per carcass size per population. The carcass size treatment was part of a separate experiment and we controlled for carcass size in the analyses we present here. We then placed each breeding box in a cupboard, and allowed parents to prepare the carcass and for the female to lay the clutch of eggs. After 53 h, and in keeping with the procedure experienced by the No Care populations [20,21], we removed both parents. We also discarded the carcasses they had prepared and buried.

We carefully sifted through the soil of each breeding box to remove the entire clutch of eggs. We placed each clutch onto a petri dish containing a 1.5% agar solution dissolved in phosphate buffered saline. Petri dishes were kept in darkness, and we checked for newly hatched larvae every 4 h starting at 56 h after pairing. We removed larvae from the petri dishes at each check to ensure that they did not damage the other eggs. After 110 h of checking, we considered any remaining eggs as hatching failures.

### (e) Statistical analysis

We were interested in two different aspects of the hatching pattern of a clutch of eggs, each a different measure of hatching synchrony, namely, hatching spread and hatching skew. Hatching spread is defined as the time that elapsed between the hatching of the first and last larvae from a given clutch [26,27]. A shortened hatching spread means that larvae are more likely to interact with each other when gaining access to the carrion nest. We predicted this would be advantageous in the No Care populations. Therefore, we compared egg-hatching spread in clutches laid by Full Care Control and No Care females. We analysed the data with a linear mixed model, with hatching spread as the response variable. We controlled for carcass size (small or medium) statistically by including it as an explanatory variable. Population type (Full Care Control or No Care) was also an explanatory variable. Initial models included the interaction between carcass size and population, but this was removed from the model as it did not significantly explain any variation (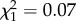, *p* = 0.79). Clutch size was included as a covariate. We had two replicate populations per treatment in experimental evolution work. Therefore, we included Block as a random effect to account for variation between the replicate populations. The significance of terms was assessed with a likelihood ratio test after systematically removing each variable from the model.

The second aspect of the hatching pattern, hatching skew, describes the extent to which hatching is skewed towards earlier or later, or evenly distributed, in the hatching spread. While egg-laying by *N. vespilloides* females can last up to 53 h, more eggs are laid earlier than later [27,32], such that hatching is naturally skewed towards the earlier part of the hatching spread. Thus, hatching spread may not properly estimate the extent of synchrony in hatching for the majority of the clutch. We calculated the hatching skew of clutches laid by Full Care Control and No Care females, and compared the measures between treatments. The greater the extent of this skew, the more synchronous is the overall hatching pattern within the spread. To estimate hatching skew, we used the hatching skew index, *V_t_*, presented in ref [23], which is given as:3.2
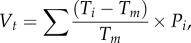
where *T_i_* is specific time interval, *T_m_* is the middle of the hatching period, and *P*_i_ is the proportion of larvae that hatched at *T_i_*. A hatching skew index of 0 would indicate that hatching was evenly distributed across the hatching period, whereas a hatching skew index of −1 would indicate that hatching was highly skewed towards the early part of the hatching period. This metric is relatively robust to outliers as the hatching of eggs at each time step is relative to the proportion of eggs that hatched in total. We performed a linear mixed model with hatching skew as the response variable. Population and carcass size class, and their interaction, were included as explanatory variables. Clutch size was included as a covariate. Block was included as a random term. We used the lme4 package [33] in R 3.4.3 [30] for the analyses. Diagnostics were performed for all analyses, and all models conformed to parametric assumptions. No eggs at all hatched in three clutches and these clutches were consequently removed from the analysis, resulting in a final sample size of 117 clutches.

## 3. Results

### (a) Experiment 1: Is hatching ‘synchrony’ adaptive in the absence of parental care?

**Figure 1.**
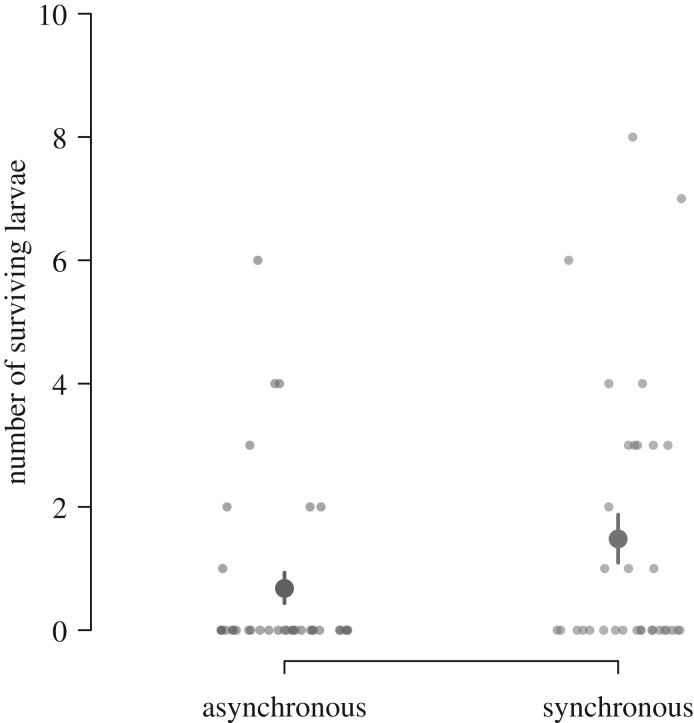
The number of surviving larvae in relation to the degree of hatching synchrony when there was no post-hatching parental care. Ten larvae were added to a carcass, either at the same time (simulating synchronous hatching) or over 28 h (simulating asynchronous hatching). Failed broods had zero larvae surviving. Means and standard errors are shown. (Online version in colour.)

Synchronous broods were more likely to produce surviving larvae than asynchronous broods in the absence of parental care (figure 1, z = −2.01, *p* = 0.044). However, continuous variation in the extent of synchrony did not affect the number of surviving larvae from a brood (*z* = 0.89, *p* = 0.38). Irrespective of the synchrony manipulation, we found that broods were more likely to fail on larger carcasses (*z* = −3.25, *p* = 0.001). However, among those broods that yielded at least one surviving larva, carcass size did not affect the number of surviving larvae (*z* = 0.64, *p* = 0.52).

Consistent with our previous work [16], and with the hypothesis that the presence of siblings is beneficial to larvae that do not have post-hatching parental care, we found that larval mass at dispersal increased with brood size (*t* = 2.74, *p* = 0.008). The synchrony manipulation did not influence the mass attained by larvae at dispersal (*t* = −0.85, *p* = 0.40), nor did the mass of the carcass (*t* = 1.64, *p* = 0.11).

### (b) Experiment 2: Has egg-hatching evolved to be more synchronous in the absence of parental care?

**Figure 2.**
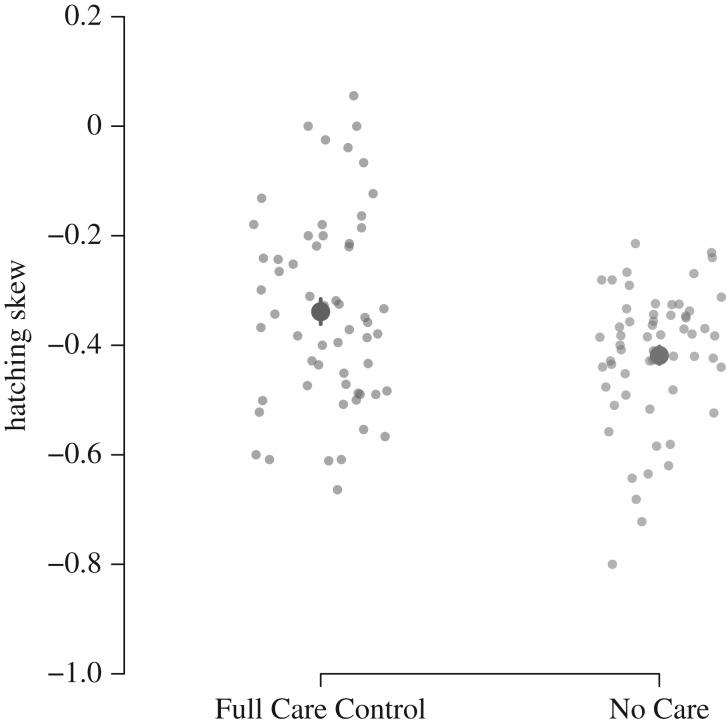
The extent of hatching skew in the No Care and Full Care Control experimentally evolving populations. A negative skew indicates that more larvae hatched earlier in the hatching period. Each point represents a different brood. Means and standard errors are shown. (Online version in colour.)

Hatching was skewed to occur significantly earlier in the hatching period in the No Care populations than in the Full Care Control populations (figure 2, 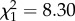, *p* = 0.004). This difference in hatching skew was independent of the size of the clutch (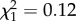, *p* = 0.73), although there was a non-significant trend towards an effect of carcass size (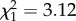, *p* = 0.07) which arose because egg-hatching on smaller carcasses was skewed more towards the early hatching period.

**Figure 3.**
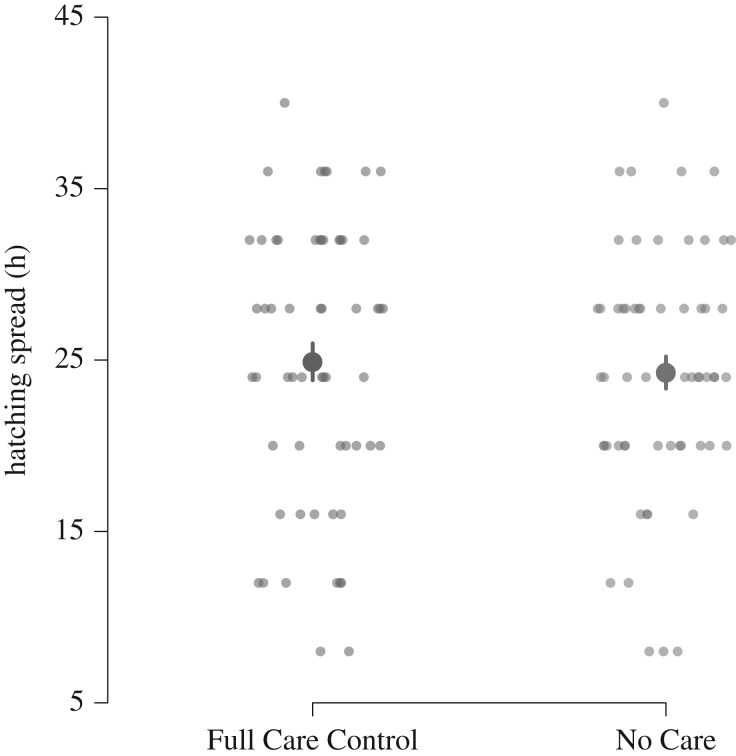
The extent of hatching spread in the No Care and Full Care Control experimentally evolving populations. Hatching spread is defined as the time which has elapsed between the first and last hatched eggs. Each point represents a different brood. Means and standard errors are shown. (Online version in colour.)

Hatching spread did not differ between the Full Care Control and No Care populations (figure 3, 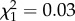, *p* = 0.85). Hatching spread was greater when eggs were laid by females that bred on a larger carcass (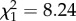, *p* = 0.004), but the effect of carcass size on hatching spread did not differ between populations (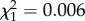, *p* = 0.94). Hatching was also spread over a longer period when clutches were larger (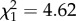, *p* = 0.03).

## 4. Discussion

Our experiments demonstrate that parents can promote offspring survival, without providing post-hatching care, by ensuring that most of their eggs hatch at a similar time. We further show that populations can adapt to a sustained loss of parental care by evolving clutches in which egg-hatching is better synchronized.

Greater hatching synchrony most likely promotes larval survival because it causes larger teams of larvae to arrive at the carcass nest at the same time, generating teams that work together more effectively to penetrate the carcass and gain access to the resources upon it [16]. This cooperative effort is probably achieved through multiple sets of mandibles working simultaneously on the carcass (Jarrett *et al*., in press [31]). Larvae that fail in this task die within 8 h of hatching [24]. Since asynchronous hatching reduced the group sizes of larvae initially arriving at the carcass, they were less likely to successfully penetrate the carcass, imperilling later hatching larvae who were also in small teams and therefore less capable of gaining access to the carcass themselves. Thus, our results suggest that the fate of the entire brood is connected to the ability of the first-hatched larvae to successfully open up the carcass. If the team was larger, and was successful, the brood survived. If it was smaller and failed to open the carcass, the whole brood was more likely to die. We think this explains why the extent of simulated hatching synchrony influenced brood survival but not the number of surviving larvae.

When we analysed the extent of hatching synchrony in populations evolving with and without post-hatching parental care, we found that hatching was skewed so that more larvae hatched earlier in the No Care than in the Full Care Control populations. Since the results of our first experiment show that this can promote brood survival in the absence of parental care, we conclude that there has been adaptive change in this trait, over 22 generations of experimental evolution. The timing of egg-hatching is explained in part by the timing of egg-laying by the mother [26] and probably also by genes that control development time in the offspring [34]. We found no evidence that females achieve better synchrony in egg-hatching simply by laying larger clutches. Egg-laying occurs during carcass preparation, when males and females work together to shave the carcass, roll it into a ball, smear the flesh with exudates, and bury it [35]. Our findings suggest that females in the No Care populations have now evolved to lay most of their eggs earlier during carcass preparation than females in the Full Care Control populations, although this interpretation remains to be tested explicitly in future work.

What prevents females in the Full Care Control populations from doing this, too? One possibility is that the timing of egg-laying is constrained by the activities connected with carcass preparation. Females in the No Care populations may have released themselves from these constraints by transferring more of these duties to the males [35]. A different possibility is that egg-laying and carcass preparation are jointly very costly and together constrain the level of care that can be supplied after hatching [36]. Females in the No Care populations are liberated from such costs because they no longer have an opportunity to care for their offspring after hatching. A final possibility is that the timing of egg-laying is determined not only by costs but also by the benefits that females stand to gain. If parents are guaranteed to be present after hatching then they can help their offspring gain access to the carcass, and the teams of earliest hatching larvae need not be very large. Egg-hatching can then take place at regular intervals, rather than being skewed towards earlier hatching. Note that none of these three suggestions is mutually exclusive and all remain to be tested properly in future work. If the third of these suggestions scales from populations to species, then we would expect to see that burying beetle species which obligately supply care to their young also exhibit a more even pattern of egg-hatching. By contrast, species of burying beetle which facultatively supply post-hatching care should have clutches skewed towards earlier hatching. There are too few data available to test this idea formally, but two species with facultative care, *N. vespilloides* [27] and *N. quadripunctatus* [37,38], each have hatching patterns that are pronouncedly skewed towards earlier hatching.

A broader implication of our findings, which extends to other species, is that offspring can act as surrogates for caring parents, as long as they act cooperatively rather than competitively. Indeed, in some insect taxa without parental care, siblings take on duties that are commonly assumed by parents in other species: they defend each other from attack by predators [2] and feed one another, too [6]. Framed like this, the puzzle is to explain why parents should bother to take on any duties of care when they could simply leave broodmates to take care of themselves, especially as broodmates can gain inclusive fitness benefits by providing sibling care. We think there are two broad solutions to this puzzle. The first is that parents will care for offspring when they can enhance the fitness of offspring in ways that siblings cannot. By being larger, parents might be better able to defend offspring from attack, for example. By being older, parents can also pass on key resources to offspring that siblings have not yet acquired such as symbionts [39], or antibodies [40], or food that cannot be acquired by developing offspring, or a territory, or skills that can only be acquired by social learning. This solution resonates with previous analyses of the conditions that favour the evolution of parental care (reviewed by [41]). The second general solution, suggested by the work we present here, is that it takes a team of offspring to replace the work of one or two parents. Therefore, unless parents can afford to produce many offspring per brood, it will be more efficient for them to provide care themselves. This hypothesis predicts that broods should evolve to be smaller when parents supply care, but larger when they do not. In support of this prediction, Gilbert & Manica found that in insects smaller broods are associated with the supply of parental care [42]. This hypothesis also explains why transitions from no care to parental care are more frequent than transitions from parental care to no care (e.g. [43]): parents can easily replace and surpass the quality of care provided by broodmates, whereas the reverse is much less likely to be true.

In summary, we have shown that synchronous hatching enables offspring to help each other when parents are not present to provide care. Whereas asynchronous hatching in birds is known to promote competitive asymmetries among broodmates, synchronous hatching in insects appears more likely to facilitate offspring cooperation in the absence of parental care. We have also shown that greater synchronicity in offspring-hatching evolves in response to the loss of post-hatching care. However, we suggest that a team of broodmates interacting cooperatively can seldom be as beneficial as the provision of care by parents and is therefore unlikely to compensate fully for the loss of parental care.

## Data accessibility

Data are deposited in figshare (doi:10.6084/m9.figshare.6938459).
